# Diagnostic tools should be used for the diagnosis of chemotherapy induced peripheral neuropathy in breast cancer patients receiving taxanes

**DOI:** 10.1002/cnr2.1577

**Published:** 2021-10-23

**Authors:** Frank van Haren, Sandra van den Heuvel, Mandy Ligtenberg, Kris Vissers, Monique Steegers

**Affiliations:** ^1^ Department of Anesthesiology, Pain and Palliative Medicine Radboudumc Nijmegen The Netherlands

**Keywords:** chemotherapy, CIPN, diagnostic tool, polyneuropathy, taxane

## Abstract

**Background:**

Though the incidence, characteristics, and pathogenesis of chemotherapy induced peripheral neuropathy (CIPN) by taxane based chemotherapy were extensively studied, diagnostic guidelines extent only recently.

**Aim:**

To observationally investigate whether specific tests can be used to predict and monitor CIPN severity.

**Methods:**

Fourteen female breast cancer patients receiving paclitaxel or docetaxel were evaluated using the McGill Pain Questionnaire (MPQ), National Cancer Institute Common Toxicity Criteria (NCI‐CTC) grading, clinical total neuropathy score (TNSc), quantitative sensory testing (QST) of pressure pain threshold (PPT), and numeric rating scale (NRS) scores and stocking and glove distribution testing (SGDT), at the start (T0), midst (T1), and end (T2) of their treatment and after 3 months (T3).

**Results:**

At T3, patients scored NCI‐CTC neuropathy grade 1 (14.3%), 2 (64.3%), and 3 (14.3%) respectively. Fifty percentage scored at least grade 1 at T0, with complaints *not* caused by CIPN. Pain, if present, was denominated “tingling” and “cold” in the MPQ. Median TNSc score increased from T0 (2.43) to T1 (4.71) to T2 (5.50) to T3 (5.57), as did pinprick and cold sensation disturbances in SGDT. PPT and associated NRS remained unchanged. TNSc and SGDT at T1 could not predict the NCI‐CTC grade at T3.

**Conclusion:**

NCI‐CTC, TNSc, and stocking and glove distribution testing can be used in the early diagnosis and monitoring of CIPN, with false‐positive findings at baseline. Final NCI‐CTC grades could not be predicted.

## 1 INTRODUCTION

Yearly, 1.7 million women are diagnosed with breast cancer worldwide. Treatment can comprise (a combination of) surgery, radiotherapy, chemotherapy, monoclonal antibody therapy, and hormone blocking therapy, depending on disease stage and specific gene expression.^1^ To improve survival, anthracycline and cyclophosphamide therapy is often combined with a taxane.^2^ Chemotherapy induced polyneuropathy (CIPN) is a commonly reported side effect (incidence up to 68.1%), prompting dose reduction or discontinuation, thus worsening outcome.^3^, ^4^

While a good target for antineoplastic drugs, microtubules (intracellular structures involved in movements in eukaryotic cells and in mitosis) play a role in the pathogenesis of CIPN by taxanes (and vinca alkaloids).

Usually they are in a state of dynamic instability, a process dependent on (de)polymerization during which the ends of microtubules switch between phases of growth and shortening.^5^ Taxanes prevent the depolarization of microtubules, thus impeding normal dynamic instability essential for cell division in malignant cells (hence: “microtubule stabilizing agents”), and microtubule‐based axonal transport. They also cause macrophage activation in dorsal root ganglia and peripheral nerves and microglial activation within the spinal cord, resulting in deficits in signal transduction, progressing from distal to proximal.^6^, ^7^, ^8^

Since the introduction of taxanes, the incidence, and characteristics of CIPN have been extensively described, using different scales for grading (e.g., the World Health Organization [WHO], Eastern Cooperative Oncology Group [ECOG], Ajani and National Cancer Institute Common Terminology Criteria [NCI‐CTC] scales). All grade neuropathic symptoms between 0 (no symptoms) and 4 (severe symptoms, loss of function), grade 3 usually triggering chemotherapeutic agent dose correction.^9^, ^10^, ^11^, ^12^ Despite different grading systems, diagnostic and screening guidelines are only recently build.^13^, ^14^

In this prospective and observational feasibility study we evaluate clinical tools for screening, early diagnosis and evaluation of CIPN in breast cancer, evaluating pitfalls and practicality of their application by comparing results for different tests and points in time. For broad application, tools should be easy to use, widely available and validated. Therefore, we selected questionnaires and neurological tests meeting these criteria: McGill Pain Questionnaire Dutch Language Version (MPQ‐DLV), NCI‐CTC, clinical version of the total neuropathy score (TNSc), quantitative sensory testing (QST) of the pressure pain threshold (PPT), and stocking and glove distribution testing (SGD testing).

## 2 MATERIALS AND METHODS

### 2.1 Study design and setting

For this prospective observational cohort study, patients were recruited and assessed in the Alexander Monro breast cancer institute's outpatient clinic in Bilthoven between February 2017 and October 2017.The study protocol was reviewed and approved by the regional ethics committee (CMO Regio Arnhem‐Nijmegen). Written informed consent was obtained from all patients for participation in the study and publication of anonymous data. Standard care was provided. The STROBE statement checklist for observational studies (cohort) was followed if applicable. The data that support the findings of this study are available from the corresponding author upon reasonable request.

### 2.2 Patients

Of 21 screened women, we included 16 female patients diagnosed with of breast cancer scheduled for treatment with taxane based chemotherapy, aged 18 years or older. Patients with a history of chronic pain, pre‐existing neuropathy, a history with risk factors for polyneuropathy (e.g., alcohol abuse, diabetes mellitus, vitamin B12 deficiency) or not able or willing to give informed consent, were excluded.

#### 2.2.1 Measurements

Fourteen out of sixteen included subjects were interviewed and neurologically assessed four times during chemotherapy cycle: at the start (T0), midst (T1), and end (T2) and after 3 months (T3). At baseline, age, ethnicity, medical history, height, weight, current medication, tumor characteristics, and treatment characteristics were recorded. At each assessment point patients filled out the MPQ‐DLV, the NCI‐CTC grade and TNSc were determined, and QST of PPT and SGD testing took place. A NCI‐CTC grade of CIPN at T3 was considered the final outcome of severity. For the further tests, their predictive value at earlier stages for the NCI‐CTC grade at T3, and thus long‐term severity, was investigated.

#### 2.2.2 National Cancer Institute Common Toxicity Criteria

The Indication for Common Toxicity Criteria Grading of Peripheral Neuropathy Questionnaire (ICPNQ) was used to determine the severity of CIPN on the NCI‐CTC scale, grading 1 (sensory changes or pain), 2 (any autonomic symptoms or subjective motor weakness), 3 (symptoms lead to ADL impairment), or 4 (ADL dependency).^15^, ^16^

#### 2.2.3 McGill Pain Questionnaire Dutch Language Version

The McGill pain questionnaire consists of 20 groups of words, each containing three to four adjectives used to describe pain sensations, of which patients choose a maximum of 1 per group. The adjectives can be categorized in one of three dimensions of pain: sensory, affective, or evaluative dimensions. These descriptions form the core of the MPQ, although the questionnaire also contains questions about the influence of the pain on quality of life (QoL), visual‐analogue scale (VAS) for pain intensity, and questions about location and course of the pain. To obtain insight in pain characteristics (if present), only the list descriptive words from the MPQ‐DLV, was used.^17^

#### 2.2.4 Total neuropathy score

The TNS has been validated against other oncological grading scales for peripheral neurotoxicity (NCI‐CTC, Eastern Cooperative Oncology Group [ECOG], and Ajani's). The shortened TNSc, used in this study, includes evaluation of sensory symptoms, motor symptoms, autonomic symptoms, pinprick sensibility, vibration sensibility, strength, tendon reflexes, scoring 0–4 on 7 items. It has a positive correlation with the NCI‐CTC grading system and an even higher sensitivity to CIPN changes.^18^ The full TNS also scores vibration sensation (QST of vibratory detection threshold) and nerve conduction studies (sural and peroneal amplitudes), thus scoring 0–4 on 10 items.

Severity of sensory, motor, and autonomic symptoms were evaluated by asking patients for localization of symptoms, extent of limitation and number of symptoms, respectively. Pinprick sensibility was tested in the upper and lower extremities. Vibration sense was tested by placing a vibrating tuning fork (128 Hz according to Hartmann, Rudolf Riester GmbH, Jungingen, Germany) on bony prominences of the upper (distal interphalangeal and proximal interphalangeal joints of the index finger, ulnar styloid process, olecranon) and lower extremities (first metatarsophalangeal joint, lateral malleolus, patella), bilaterally. If vibration sense in the most distal testing point was not disturbed, more proximal points were not tested and assumed to be normal (for TNSc: reduced in fingers/toes, up to wrist/ankle or up to/ above elbow/knee).^19^ Strength of extension and flexion of the hands and feet was evaluated according to the Medical Research Council scale. Last, the deep tendon reflex of the left and right Achilles tendons was tested. If the Achilles tendon reflex was reduced or absent, the patella tendon reflex was tested as well.

#### 2.2.5 Quantitative sensory testing

Patients with neuropathic pain often develop mechanical hyperalgesia.^20^ By testing the PPT in our patients, we explore if the same is true for patients with CIPN. PPT was measured with the Wagner Force Ten™ FDX digital force gage; a handheld pressure algometer. The pressure was exerted on the left and right musculus trapezius pars medialis (4 cm paravertebral of vertebrae T3), musculus rectus femoris (10 cm proximal to patella), thenar eminence (on the middle of the muscle) and musculus abductor hallucis (on the middle of the muscle). Patients were instructed to tell the researcher to stop applying pressure if it became too uncomfortable or painful. The applied pressure in kilopascal (kPa) was then recorded. Patients were asked to score pain according to the Numeric Rating Scale (NRS).

#### 2.2.6 SGD testing

Sensory CIPN symptoms in patients treated with taxanes often emerge in a stocking and glove pattern.^20^, ^21^ To assess sensory changes, cold sensibility was measured delivering stimuli with an ice cold Tip‐Therm and pinprick sensibility was measured delivering stimuli with a BD™ 18 G Blunt Fill Needle. Stimuli were given every 3 cm from the cubital fossa to the top of the third digit and from the patella to the top of the first (big) toe on the left and right side. Patients were instructed to indicate changes in sensation (hypo‐ or hyperalgesia) along the testing points. If present, the distance (cm) between the beginning and the end (often being the tip of digit III or the great toe) of the area with changed sensibility was recorded.

### 2.3 Statistical analysis

Statistical analyses were performed using IBM SPSS Statistics 22 for Windows. Descriptive statistics were used to describe patient characteristics and to report the results of the MPQ, NCI‐CTC grades and SGD testing, resulting in number of cases (*n*) and mean ± SD/median–interquartile range (range) for averages. Non‐continuous changes in NCI‐CTC, TNSc, and NRS were assessed with the Friedman test and *χ* ^2^. Mean PPT and maximum NRS were used for each anatomical site at any point in time, and the length of the affected area (left and right, upper, and lower extremity) for SGD testing and further analyzed using one‐way analysis of variance in normal distribution (Shapiro–Wilk test, *p* > .05) and sphericity (Mauchly's, *p* > .05). For prediction of NCI‐CTC grade at T3, ordinal regression analysis was used. *p* values <.05 in two‐tailed tests of significance, are considered significant.

## 3 RESULTS

Of 16 enrolled patients, 2 patients were excluded at T2 and T3 respectively because of missed appointments and patient withdrawal. Patient characteristics are shown in Table 1.

**TABLE 1:** Patient characteristics shown as number of cases (*n*) and mean ± SD/median—Interquartile range (range) for averages

Number of patients (*n*)	14
Age (years)	49.6 ± 35.65/48.5–8.0 (25–67)
Disease stage (*n*)	
T1, T2, T3	2, 11, 1
N0, N1, N2, N3	5, 6, 2, 1
Treatment with docetaxel (*n*)	9
Number of cycles	5.3 ± 1.0/6.0–2.0 (4–6)
Cumulative dose in mg m^−2^	782.2 ± 85.1/720.0–120.0 (720–960)
Treatment with paclitaxel (*n*)	5
Number of cycles	12.0 ± 0/12.0–0.0 (n/a)
Cumulative dose in mg m^−2^	1560 ± 254.6/1560–360 (1200–1920)
Treatment prior/concomitant to taxane (*n*)	
Surgery	14
Radiotherapy	10
Monoclonal antibodies therapy	8
Other chemotherapeutic agents	
Anthracyclines and cyclophosphamide	10
Carboplatin	4

Dose reduction was required in six subjects due to toxicity, including severe neuropathy in two cases. In one of these two patients, taxane therapy was reduced by two cycles due to the neuropathy symptoms. We ran two sets of analysis of data—first to compare the outcomes at T0, T1, and T2 (complete study population) and then a second analysis to compare the outcomes of the patients tested at T3 with their previous outcomes (within subject comparison).

### 3.1 National Cancer Institute Common Toxicity Criteria

**FIGURE 1:**
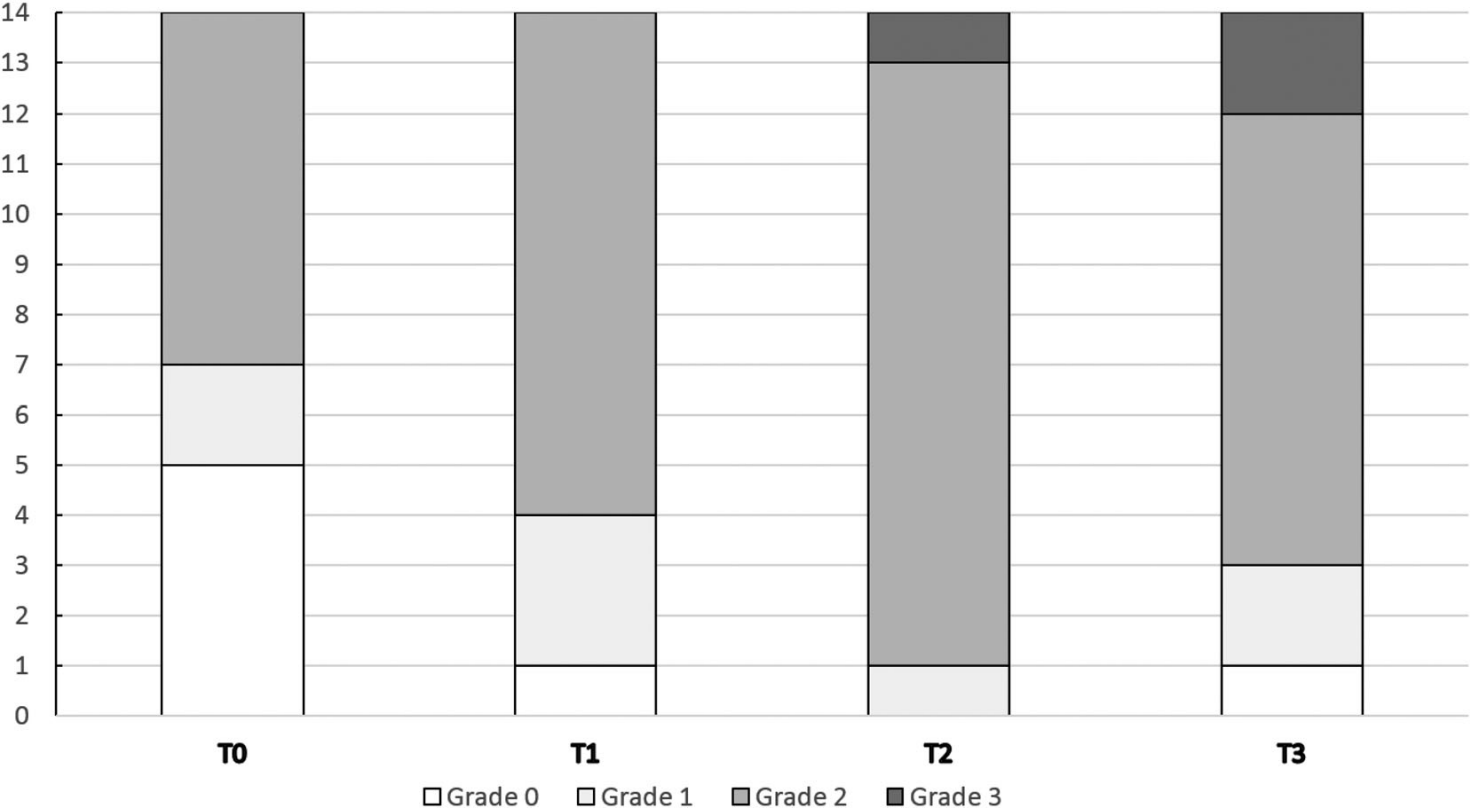
Distribution of National Cancer Institute Common Toxicity Criteria (NCI‐CTC) grades at T0, T1, T2, and T3. Results shown as numbers of cases for NCI‐CTC grade at T0, T1, T2, and T3 respectively. Distribution among the total of 14 cases is presented

CIPN grade distribution at each measuring time are shown in Figure 1. According to the ICPNQ questionnaire, nine patients already had peripheral neuropathy grade 1 (*n* = 7) or 2 (*n* = 2) at T0. Complaints of sweating (*n* = 3) were reported to be related to previous treatment with Doxorubicin and Cyclophosphamide (*n* = 2) and/or were present only locally after breast‐ and axilla surgery (*n* = 2). The scoring of urinary disfunctions (*n* = 3, previously existing problems with urinary continence), sensory changes (*n* = 2, unilateral tingling sensations in one hand after surgery or foot, previously existing palpitations (*n* = 1) and positional dizziness related to low blood pressure and/ or low hemoglobin levels (*n* = 1) were all unrelated to (CI)PN.

At T1 two of the patients suffered of grade 1 neuropathy, 10 patients of grade 2 neuropathy, and the remaining two patients had no neuropathy symptoms. Grade 2 neuropathy was mainly caused by autonomic symptoms as bladder dysfunction, palpitations, increased sweating and subjective weakness. Next to autonomic symptoms, patients mentioned the following sensory symptoms: dull feeling, tingling, and pain in upper and lower extremities.

At T2 all patients experienced (substantively comparable) symptoms, of which the majority a grade 2 neuropathy (*n* = 10). One patients with grade 3 suffered from bladder dysfunction, constipation, and increased sweating and a dull feeling and pain in extremities, impairing ADL.

At T3 all but one patient suffered from neuropathy. Two had grade 1 neuropathy, nine grade 2, and two grade 3. At T3 subjective weakness became more prominent, especially in the hands, which led to impairment in activities of daily life (ADL) in some patients. Remarkably, the one patient with no neuropathy at T3 still had grade 2 neuropathy at T2. In all other patients no recovery was seen, their complaints either remained stable or worsened.

For NCI‐CTC there is a significant difference when comparing scores at T0 and T3 (*χ* ^2^(2) = 17.105, *p* < .001). Post‐hoc analysis with the Wilcoxon signed‐rank test showed a significant difference from T0 (Mdn = 0.00) to T1 (Mdn = 2.00) (*z* = −2.879, *p* = .004), from T0 to T2 (Mdn = 2.00) (*z* = −3.127, *p* = .002) and from T0 to T3 (Mdn = 2.00) (*z* = −2.701, *p* = .007), but not between T1, T2, and T3 (*p* > .05).

### 3.2 Total neuropathy score

TNSc score was statistically significantly different at the various time points when comparing the outcomes at T0–T2 of the complete study population (*χ* ^2^(2) = 15.148, *p* = .001), as well as when comparing T0–T3 outcomes in the already tested subjects (*χ* ^2^(3) = 19.324, *p* < .0005). Mean TNSc score ± SD (range) was 2.43 ± 1.09 (1–4), 4.71 ± 2.27 (1–8), 5.50 ± 2.68 (2–12), and 5.57 ± 2.53 (2–10) for T0, T1, T2, and T3 respectively.

Post hoc analysis with the Wilcoxon signed‐rank test revealed statistically significant differences in TNSc score between T0 and T1 (*z* = −2.955, *p* = .003), T2 (*z* = −3.163, *p* = .002), and T3 (*z* = −2.806, *p* = .005), but not between T1, T2, and T3 (*p* > .05). Since the TNSc showed a significant difference when comparing T0 to T1, but there were no significant differences in TNSc between T1, T2, and T3, we used ordinal regression to test whether the TNSc score at T1 (as early as possible) could be related to NCI‐CTC‐scores at T3.

### 3.3 NCI‐CTC versus TNSc

The correlation between NCI‐CTC grade and TNSc score was 31.8% (*p* = .017) for all 56 measurements. Graphical representation of the different measurements (14 patients at 4 time points) in Table 2.

**TABLE 2:** Distribution of NCI‐CTC grade versus TNSc score

NCI‐CTC grade	12				1	
	11					
	10				1	
	9					
	8			3		
	7		2	6	1	
	6			6		
	5		1	4		
	4	3		3		
	3	1	4	4		
	2	3	1	7		
	1			4		
	0					
		0	1	2	3	4
		TNSc score				

*Note*: Results shown as numbers of cases for all measurements.

Abbreviations: NCI‐CTC, National Cancer Institute Common Toxicity Criteria; TNSc, total neuropathy score.

### 3.4 McGill Pain Questionnaire Dutch Language Version

At T0 three patients indicated to experience pain: two described it as “tingling” (left hand and right foot respectively) and one as “pulling” (left hand). At T1 again three patients had pain; the two patients with the “tingling” feeling still among them, although one now described the pain as “cold.” The patient with the “pulling” pain at T0 had no pain at T1. One patient had new onset of pain in the feet, described as “mild” and “pricking.” At T2 seven patients reported pain, mainly described as “tingling” and “tight.” At T3 5 out of the 11 patients still had pain, predominantly described as “tingling” and “cold.”

### 3.5 Quantitative sensory testing

For all measurements in one specific subject PPT could not be found, because it was above maximum pressure. This patient was excluded from further analysis of PPT and NRS. Analysis with non‐parametric Friedman testing in the presence of multiple outliers, presented median PPT and NRS scores at the different measuring points and times, showed none of the QST measurements of PPT (at any measuring point and measuring time), as well as NRS‐scores, showed significant changes over time. Graphic presentation of these results are presented in Table 3.

**TABLE 3:** PPT and NRS scores at different muscles at T0, T1, T2, and T3 (*n* = 14), shown as mean ± SD (range)

	T0	T1	T2	T3
*Trapezius muscle*				
PPT	55.9 ± 27.5 (19.0–12.0)	53.2 ± 20.9 (15.2–98.0)	53.7 ± 23.8 (13.2–111.4)	55.6 ± 17.3 (32.2–92.6)
NRS	5.29 ± 1.82 (2.0–8.00)	5.43 ± 1.79 (2.0–8.0)	5.36 ± 1.79 (2.0–8.0)	5.32 ± 2.11 (0.0–8.0)
*Rectus femoris muscle*				
PPT	75.5 ± 25.3 (22.4–119.6)	73.2 ± 25.0 (17.0–125.2)	77.7 ± 27.0 (17.0–151.4)	81.4 ± 27.0 (36.0–141.8)
NRS	4.96 ± 1.93(2.0–9.0)	4.82 ± 2.65 (0.0–9.0)	5.11 ± 2.18 (1.0–8.0)	4.64 ± 2.67 (0.0–8.0)
*Thenar muscle*				
PPT	51.6 ± 23.0 (22.6–111.8)	40.3 ± 34.73 (18.8–109.2)	48.4 ± 20.14 (11.2–94.8)	48.7 ± 12.7 (30.2–73.8)
NRS	5.43 ± 1.62 (2.0–8.0)	5.30 ± 2.09 (0.0–8.0)	5.64 ± 1.39 (3.0–7.0)	5.43 ± 1.95 (2.0–8.0)
*Abductor hallucis muscle*				
PPT	54.0 ± 21.2 (19.0–90.8)	51.0 ± 16.6 (18.8–77.6)	51.8 ± 19.5 (18.8–92.8)	46.0 ± 13.43 (19.8–70.0)
NRS	5.60 ± 1.62 (2.0–8.0)	5.61 ± 1.69 (2.0–8.0)	5.25 ± 1.86 (2.00–8.00)	5.25 ± 2.30 (0.0–8.0)

### 3.6 SGD of pinprick and cold testing

**FIGURE 2:**
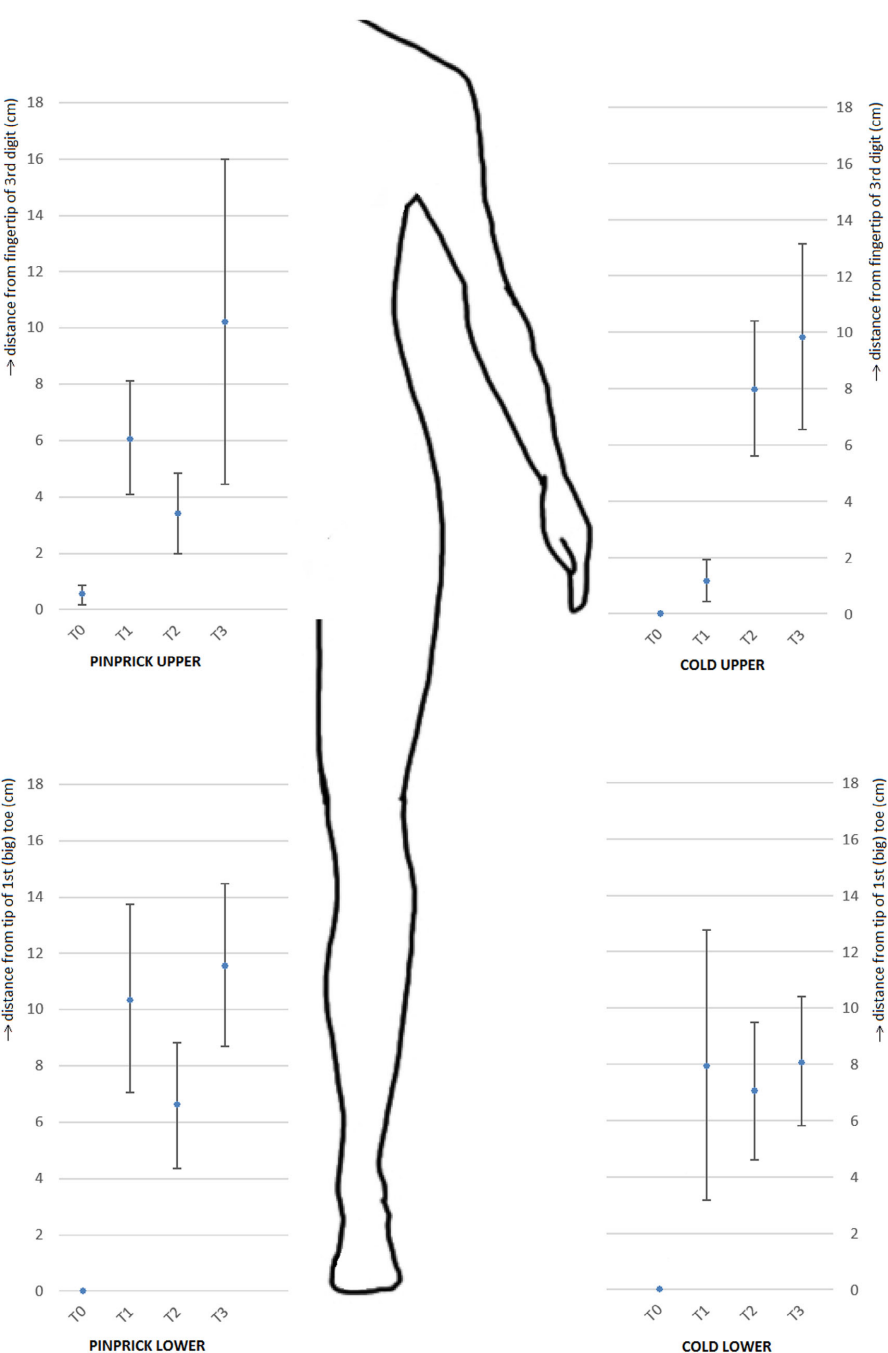
Mean length (cm) of the affected areas as assessed by stocking and glove distribution (SGD) testing for pinprick and cold testing (“pinprick” and “cold” respectively) of the upper and lower extremities (“upper” and “lower” respectively)

Paresthesia usually starts at the most distal nerve endings (fingers and toes) and progresses proximally.^4^ We found this same pattern of distribution in our patients: 1 at T0, 7 at T1, 9 at T2, and up to 11 of the tested patients at T3. Our patients complained approximately two times as often of hyperalgesia than hypoalgesia. Paresthesia appeared in a symmetrical pattern in all except one case. In three cases we found areas of hypo‐ or hyperalgesia for pinprick or cold sensation, but not in a stocking and glove distribution (e.g., 10 cm hypoalgesia in the forearm when testing pin prick sensation, but no sensory disturbances in de hand or fingers). The analyzed length of the affected areas over time are shown in Figure 2.

Friedman testing in the presence of multiple outliers and abnormal distribution, presented significant differences in outcomes for pinprick in upper (*χ* ^2^ = 9.14; *p* < .05) and lower extremities (*χ* ^2^ = 21.29; *p* < .01) and cold sensation in upper (*χ* ^2^ = 17.08, *p* < .01) and lower extremities (*χ* ^2^ = 17.04; *p* < .01) at T0, T1, and T2 for the complete study population. Results of post‐hoc Wilcoxon signed‐rank testing are presented in Table 4.

**TABLE 4:** Analysis of stocking and glove distribution (Wilcoxon Signed‐Rank, Z[*p* value])

	Pinprick		Cold testing	
	Upper	Lower	Upper	Lower
T0–T1	−2.68 (<.01)*	−2.67 (<.01)*	−1.63 (.102)	−2.37 (<.05)*
T0–T2	−1.95 (.051)	−2.94 (<.05)*	−2.67 (<.01)*	−2.94 (<.01)*
T0–T3	−2.32 (<.05)*	−3.73 (<.01)*	−3.06 (<.01)*	−3.31 (<.01)*
T1–T2	−1.02 (.307)	−1.40 (.162)	−2.59 (<.01)*	−1.26 (.207)
T1–T3	−0.17 (.864)	−0.65 (.513)	−2.69 (<.01)*	−1.13 (.257)
T2–T3	−0.70 (.484)	−2.02 (<.05)*	−0.45 (.653)	−0.26 (.793)

*Statistically significant.

## 4 DISCUSSION

This study shows that NCI‐CTC grading (as determined with ICPNQ) and TNSc were both able to find significant differences compared to baseline from T1 onwards. Thus with NCI‐CTC and TNSc early signs of CIPN can be identified. However, both scoring systems were unable to detect differences between T1, T2, and T3. So either there were no actual changes in CIPN severity after T1, or the tests were not sensitive enough to track subtle changes. Only recently, guidelines are published addressing the (early) diagnosis and evaluation of CIPN, partially due to difficulties interpreting scores in the presence of co‐morbidity, differences between scores (invasiveness, objective vs. subjective, et cetera).^18^, ^22^, ^23^, ^24^

Additionally, nine patients showed signs of neuropathy according to NCI‐CTC at baseline, although pre‐existent neuropathy had been excluded. These patients scored “autonomic” symptoms related to co‐morbidity or earlier treatments in the absence of typical primary symptoms of CIPN, such as sensory disturbances. Therefore, the NCI‐CTC‐grading of CIPN using ICPNQ is not specific enough as a baseline denominator. In accordance to this, Beijers et al. showed that the sensory and motor subscales of the ICPNQ had adequate internal consistency, but the autonomic subscale did not.^16^ Not only the evaluation of autonomic symptoms, but also the evaluation of motor symptoms should be treated with caution. The presence of confounding factors (e.g., fatigue or depression), can make de judgment of motor symptoms difficult and examiners tend to overestimate the occurrence of motor neuropathy without a formal neurological examination.^22^, ^24^ Hence, only sensory symptoms such as “tingling sensation” or “pain,” can consistently be evaluated with the use of ICPNQ and NCI‐CTC.

The use of TNS(c) with neurological examination, can offer a solution in this motor symptoms misinterpretation. As mentioned, TNS and TNSc are more sensitive for CIPN changes than NCI‐CTC, as it incorporates objective and subjective measures.^17^ However, we encountered some difficulties when performing TNSc. At baseline we found TNSc scores ranging from 0 to 4 (out of 28) in our patients. All the scores >0 at baseline were caused due to decreased or absent ankle jerks and/or deficits in vibratory sensation, that could partly be explained by inter‐rater variability.

QST measurements of PPT at the trapezius muscle, rectus femoris muscle, thenar eminence, and abductor hallucis muscle could not find significant changes between measuring points. Pain sensitivity for mechanical stimuli (blunt pressure) did not change. However, some caution regarding the reproducibility of a handheld pressure algometer is in place, considering that pressure must be applied at the exact same spot in all patients and at all points in time. Lacourt et al. found that first‐measurement should be excluded as they yield higher thresholds compared to following measurements,^25^ despite the good inter‐rater variability in other studies.^26^ Others factors could bias results, such as previous myalgia and varying and stress related muscle tension. Therefore, it seems that QST measurement of PPT has a limited place in the diagnosis of CIPN.

SGD testing proved useful in the diagnosis of CIPN, as it was capable to detect significant differences compared to baseline and between other time points. It could therefore be used to monitor CIPN progress. Pinprick sensation disturbances became apparent from T1 onwards, while cold sensation disturbances started later at T2. This may be explained by the fact that the perception of pinprick pain is related to activity in A‐fiber nociceptors, while cooling the skin activates not only A‐fibers ánd C‐fibers.^26^ Possibly, taxane induced neuropathy mostly affects A‐fibers. Additional research can help to determine whether this discrepancy in sensation disturbances is general, or limited to the population in this very study. Like TNSc score, SGD testing outcomes at T1 were not adequate predictors for NCI‐CTC grade at T3, according to our ordinal regression analysis.

In analysis of the subgroup of patients that also received carboplatin treatment, emerging complaints of CIPN did not occur significantly more than in other subjects. In analysis of the subgroup that also received carboplatin treatment, emerging complaints of CIPN did not occur significantly more than in the other subjects. They were therefore included in the further analysis regarding the use of diagnostic tools. Theoretically however, they could influence the different scores, by the mechanisms and specific complaints to which they cause CIPN.

Despite the small study population, this study addresses the possibilities as well as the pitfalls of different tests used in the (early) recognition and the monitoring of the progression of CIPN related complaints. For women with breast cancer receiving taxane based chemotherapy, NCI‐CTC and TNSc scales, as well as the “stocking and gloves” distribution of pinprick and cold testing abnormalities, but not (threshold) pinprick testing and associated NRS scores, are useful for determining the progression of CIPN symptoms. However, confounders influence (from baseline onwards) the definitive score. Mainly the scoring of autonomic and motor symptoms cause false positive scoring in relation to complaints caused by CIPN. These important pitfalls should be addressed when using these tests in clinical practice or research, and prevents proper guidelines to be developed for the (early) diagnosis and monitoring of CIPN. Review of the current literature, in combination with Delphi‐assessments, are useful for the formulation of an algorithm and current approach. For future research, the application to other chemotherapeutic agents, patients, and tumors and the possibility for prediction, should be addressed.

In conclusion, this feasibility study shows that diagnostic tools should be used for the diagnosis of CIPN. A definitive approach cannot be formulated, due to the influence of confounders and lack of research addressing different cancer and chemotherapy types in relation to the wide variety of available tools.

## CONFLICT OF INTEREST

The authors have stated explicitly that there are no conflicts of interest in connection with this article.

## AUTHOR CONTRIBUTIONS

**Frank van Haren:** Conceptualization (equal); formal analysis (equal); supervision (equal); visualization (equal); writing – original draft (equal); writing – review and editing (equal). **Sandra van den Heuvel:** Conceptualization (equal); data curation (equal); formal analysis (equal); writing – original draft (equal); writing – review and editing (equal). **Mandy Ligtenberg:** Data curation (equal); writing – original draft (equal). **Kris Vissers:** Conceptualization (equal); supervision (equal); writing – review and editing (equal). **Monique Steegers:** Conceptualization (equal); supervision (equal); writing – review and editing (equal).

## ETHICS STATEMENT

This study was performed in line with the principles of the Declaration of Helsinki. The study protocol was reviewed and approved by the regional ethics committee (CMO Regio Arnhem‐Nijmegen). In execution and publication of this study, the STROBE statement checklist for observational studies (cohort) was followed if applicable (included as such in Section 2). Written informed consent was obtained from all patients. Standard care was provided (included as such in Section 2).

## Data Availability

The data that support the findings of this study are available from the corresponding author upon reasonable request.
